# The anti-*Staphylococcus aureus* activity of the phenanthrene fraction from fibrous roots of *Bletilla striata*

**DOI:** 10.1186/s12906-016-1488-z

**Published:** 2016-11-29

**Authors:** Jing-Jing Guo, Bin-Ling Dai, Ni-Pi Chen, Li-Xia Jin, Fu-Sheng Jiang, Zhi-Shan Ding, Chao-Dong Qian

**Affiliations:** 1 Zhejiang Chinese Medical University, Hangzhou, Zhejiang Province China; 2 Shaoxing Central Hospital, Shaoxing, Zhejiang Province China

**Keywords:** Bletillae Rhizom, Gram-positive bacteria, Antibacterial, Time-kill assays, Postantibiotic effect

## Abstract

**Background:**

Bletillae Rhizoma, the tuber of *Bletilla striata*, has been used in Chinese traditional medicine to treat infectious diseases. Chemical studies indicated that phenanthrene was one of the most important components of the herb, with a broad spectrum of antibiotic activity against Gram-positive bacteria. The objective of this study was to further characterize the antibacterial activity of the phenanthrene fraction from the fibrous root of the pseudobulb of *B. striata*.

**Methods:**

The phenanthrene fraction (EF60) from the ethanol extract of fibrous roots of *Bletilla striata* pseudobulbs was isolated using polyamide column chromatography. The antibacterial activity of the fraction was evaluated in vitro using a 96-well microtiter plate and microbroth dilution method. The cytotoxicity of EF60 against mammalian cells was tested by hemolysis and MTT assays.

**Results:**

EF60 was obtained using alcohol extraction and polyamide column chromatography, with a yield of 14.9 g per 1 kg of the fibrous roots of *B. striata*. In vitro tests indicated that EF60 was active against all tested strains of *Staphylococcus aureus*, including clinical isolates and methicillin-resistant *S. aureus* (MRSA). The minimum inhibitory concentration (MIC) values of EF60 against these pathogens ranged from 8 to 64 μg/mL. Minimum bactericidal concentration tests demonstrated that EF60 was bactericidal against *S. aureus* 3304 and ATCC 29213 and was bacteriostatic against *S. aureus* 3211, ATCC 25923, and ATCC 43300. Consistently, the time-kill assay indicated that EF60 could completely kill *S. aureus* ATCC 29213 at 2× the MIC within 3 h but could kill less than two logarithmic units of ATCC 43300, even at 4× the MIC within 24 h. The postantibiotic effects (PAE) of EF60 (4× MIC) against strains 29213 and 43300 were 2.0 and 0.38 h, respectively. Further studies indicated that EF60 (160 μg/mL) showed no cytotoxicity against human erythrocytes, and was minimally toxic to Human Umbilical Vein Endothelial Cells with an IC_50_ of 75 μg/mL.

**Conclusions:**

Our studies indicated that EF60 is worthy of further investigation as a potential phytotherapeutic agent for treating infections caused by *S. aureus* and MRSA.

**Electronic supplementary material:**

The online version of this article (doi:10.1186/s12906-016-1488-z) contains supplementary material, which is available to authorized users.

## Background

With the widespread use of antibiotics, antimicrobial resistance has become a major medical and public health problem [1–3]. Of particular concern is methicillin-resistant *Staphylococcus aureus* (MRSA), which has disseminated throughout the world and is a global human health problem due to infections in both hospitals and the community [4, 5]. Glycopeptides are the gold standard to treat MRSA infections, but vancomycin- and teicoplanin-resistant bacteria have also emerged [6–8]. There is therefore an urgent need for new strategies to treat the infections caused by antibiotic-resistant pathogens. The discovery and development of antibacterial natural products, which are becoming increasingly popular among consumers, are an alternative method for controlling these diseases [9, 10].

Bletillae Rhizoma, the pseudobulbs of *Bletilla striata* (Reichb. f.), has been used in Chinese traditional medicine to treat pneumorrhagia and pneumonophthisis [11]. It is also frequently applied for curing skin cracks, abscesses, and burns when combined with other herbal medicine. Previous investigations on the constituents of Bletillae Rhizoma have revealed the presence of monomeric phenanthrene, dimeric phenanthrenes, and their derivatives, which contain a potent antibacterial activity against Gram-positive bacteria [12–14]. It is worth noting that phenanthrenes are a rather uncommon class of aromatic metabolites that have mainly been found in the Orchidaceae family [14]. Although a large number of phenanthrenes have been isolated from Bletillae Rhizoma and have been demonstrated to possess antimicrobial activities, further pharmacological studies of these compounds are limited due to low content of phenanthrenes in the pseudobulbs of *B. striata*.

The fibrous roots of the pseudobulbs of *B. striata* are usually discarded during its processing and commercialization, which represents a waste of natural resources. Our recent study indicated that the chemical composition of fibrous roots is similar to that of *B. striata* pseudobulbs; however, the total phenolic content in the former is higher than that in the latter [15]. Further study showed that the fibrous part of *B. striata* is a rich source of phenanthrene compounds, and six phenanthrenes, including four new biphenanthrenes containing antibacterial activity, were isolated from a 95% ethanol extract [16]. To date, 34 phenanthrene compounds isolated from *B. striata* have been extensively described [12–14, 16–18]. However, there was a predominant tendency to publish the isolation and activity screening of phenanthrenes in the past years. Little information is available on the antimicrobial activity more in depth of this kind of compounds, regardless of monomer or mixture. Thus, the aim of this study was to isolate and characterize further the antimicrobial phenanthrene fraction isolated from fibrous roots of *B. striata* pseudobulbs.

## Methods

### Bacteria strains

*S. aureus* ATCC 25923, *S. aureus* ATCC 29213, *S. aureus* ATCC 43300, *E. coli* ATCC 35218, and *P. aeruginosa* ATCC 27853 were purchased from the American Type Culture Collection. *Bacillus subtilis* 168 was a gift from Mei-Ya Li (Zhejiang Chinese Medical University, Hangzhou, China). Clinical isolates were obtained from patients at the Shaoxing Central Hospital, Shaoxing, China.

### Preparation of the phenanthrene fraction

The rhizomes of *B. striata* were collected from Tuankou Town, Zhejiang Province, People’s Republic of China, and authenticated by Prof. ZS Ding (one of the authors). A voucher specimen was deposited in Zhejiang Chinese Medical University with specimen number BS-2012-I. The air-dried and powdered fibrous roots (1.0 kg) were extracted with 15 L of 95% ethanol under reflux, three times (each time, 60 min). The extract was concentrated under reduced pressure and yielded 94 g of crude ethanol extract. The ethanol extract was loaded onto a polyamide resin column and washed with distilled water, followed by elution with 20, 40, 60, 80, and 95% (v/v) ethanol. Each fraction was collected and tested for antibacterial activity using the agar diffusion method [19]. The tests were repeated three times to ensure reliability. The active fraction (EF60) eluted with 60% ethanol in water were dried in a vacuum and analyzed using a Dionex Ultimate 3000 high-performance liquid chromatography (HPLC) System (Thermo Fisher Scientific, Waltham, USA) with a diode-array ultraviolet/visible (UV–VIS) detector. HPLC was performed using a Venusil XBP C18 (5 μm, 250 × 4.6 mm) column eluted with a gradient mixture of acetonitrile in water containing 0.1% formic acid, from 5 to 95% in 60 min.

### Determination of MIC and MBC

The minimum inhibitory concentration (MIC) was determined using a 96-well microtiter plate and the microbroth dilution method as previously reported [20, 21]. Briefly, bacteria were seeded at 2 × 10^5^ cells per well (200 μL) in a 96-well plate containing Mueller–Hinton (MH) broth (0.2% meat extracts, 1.75% acid digest of casein, and 0.15% starch) with varying concentrations of each test sample. Vancomycin and berberine were used as positive controls. Dimethyl sulfoxide (DMSO; 10 μL) and MH broth alone were used as negative controls. The MIC was defined as the lowest concentration that completely prevented visible growth after incubation at 37 °C for 18–20 h.

The minimum bactericidal concentration (MBC) was determined from tubes showing complete inhibition. A MH agar plate was seeded with 100-μL aliquots from clear tubes and incubated for 24 h at 37 °C. The MBC was defined as the lowest compound concentration resulting in a ≥3-log reduction in the number of CFU [22].

### Time-kill curves

The time-kill kinetics of antimicrobial agent against *S. aureus* ATCC 29213 and ATCC 43300 were determined [23, 24]. A logarithmic-phase broth culture of *S. aureus* was diluted in MH broth to a final count of approximately 5 × 10^5^ CFU/mL; next the antimicrobial agent was added to the broth culture to yield concentrations of 1 ×, 2 ×, and 4 × the MIC. An equivalent volume of DMSO was added to the vehicle control tube. The culture was incubated at 37 °C with shaking for 24 h. Surviving clones in each culture were determined by withdrawing samples at various time points and plating the appropriate serial dilutions onto MH agar plates.

### Effect of pH and inoculum size

The effect of changes in the pH of the medium and the inoculum size on the MIC of antimicrobial agent against *S. aureus* ATCC 29213 and ATCC 43300 were assessed [25]. The MIC was determined using the microbroth dilution method as described above. Inoculum size was remained constant in the MH broth (1 × 10^5^ CFU/mL) when the pH of the culture was adjusted to 5.0, 7.2, and 9.0 with either HCl or NaOH. In contrast, the pH value of the MH broth remained at 7.2, while the inoculum size changed to 10^3^, 10^5^, and 10^7^ CFU/mL. To prevent interference from the high inoculum concentration on the MIC, the MBCs were used to confirm the MICs. All experiments were conducted in triplicate.

### Postantibiotic effect

The Postantibiotic effect (PAE) of EF60 against *S. aureus* ATCC 29213 and ATCC 43300 was determined using MH broth. The sterilized antimicrobial agent was added to a logarithmic-phase broth culture of approximately 10^5^ CFU/mL to give concentrations equivalent to 1×, 2×, and 4× the MIC. In addition, a culture containing 5% DMSO was used as the growth control. Following 1 h of exposure at 37 °C, the antibiotic concentration was reduced via a 1,000-fold dilution into prewarmed MH broth and incubated at 37 °C for 24 h. Viable counts were measured on antibiotic-free MH broth prior to exposure and at 1, 2, 4, 6, 8, and 24 h after neutralization by dilution. The PAE was then measured according to the method previously described [26].

### Cytotoxicity assay

The cytotoxicity of EF60 against human red blood cells was assayed as previously described [27]. Human blood samples were obtained from normal volunteers. Hemolysis of red blood cells was induced by the addition of EF60, and cells were incubated for 2 h at 37 °C in 0.9% saline. The cytotoxicity of EF60 versus Human Umbilical Vein Endothelial Cells (HUVEC) was tested using a 48-h continuous 3-(4,5-dimethylthiazol-2-yl)-2,5-diphenyltetrazolium bromide (MTT) assays as previously described [28].

### Statistical analysis

Statistical analyses were performed using SPSS software (Statistical Software Package for Windows, version 19). The PAEs were expressed as the mean ± standard deviation, and differences are considered to be statistically significant at *P* < 0.05.

## Results

### Isolation of phenanthrene fractions from the fibrous roots of *B. striata*

To obtain phenanthrene fractions, ethanol extract from the fibrous roots of *B. striata* was loaded onto a polyamide resin column and eluted using step-gradient ethanol in water to generate five fractions, namely EF20, EF40, EF60, EF80, and EF95. Phenanthrenes were analyzed by analytical HPLC and identified by congruent retention times and UV–VIS spectrum, and EF60 was found to be rich in phenanthrenes (Additional file 1: Figure S1, Table S1, and Figure S2). Further study indicated that EF60 had good activity against *S. aureus* (Table 1). Thus, in the current work, subsequent efforts were focused on the characterization of this active fraction. EF60 was produced as described in the Methods section with a yield of 14.9 g per 1 kg of fibrous root.

**Table 1:** Antimicrobial activity of different polyamide resin elution fractions from *Bletilla striata* fibrous root ethanol extract

Indicator strain	Inhibition zone size (mm diameter)					
	EF20^a^	EF40^a^	EF60^a^	EF80^a^	EF95^a^	Amp^b^
*Staphylococcus aureus* ATCC 25923	NA	12 ± 0.4	21 ± 0.7	NA	NA	27 ± 0.6
*Escherichia coli* ATCC 35218	NA	NA	NA	NA	NA	20 ± 0.3

^a^Relative inhibition zone (mm) at 100 μg per paper disc

^b^Relative inhibition zone (mm) at 10 μg per paper disc

*Amp* ampicillin

*NA* no activity

### Antibacterial activity of EF60

The antimicrobial activities of EF60 were evaluated against 16 Gram-positive and 2 Gram-negative bacteria strains. As expected, EF60 was active against all the Gram-positive bacteria, with the MICs ranging from 8 to 64 μg/mL (Table 2). However, EF60 was not active against each of the Gram-negative bacteria (MIC > 128 μg/mL). Although berberine, a famous natural product from Chinese herbs, had antimicrobial activity against both Gram-positive and Gram-negative bacteria [29], its MIC against most of the strains examined was found to be >64 μg/mL (Table 2). It is worth noting that clinical isolates of *S. aureus* and MRSA ATCC 43300 were also sensitive to EF60.

**Table 2:** MIC of EF60 against standard strains and clinical isolates

Indicator strain	MIC (μg/mL)^a^		
	EF60	Berberine	Vancomycin
*S. aureus* ATCC 25923	32	128	2
*S. aureus* ATCC 29213	16	64	1
*S. aureus* ATCC 43300	8	128	2
*S. aureus* 5021	64	64	1
*S. aureus* 5022	16	64	1
*S. aureus* 5023	32	128	1
*S. aureus* 5024	64	256	2
*S. aureus* 5025	16	128	1
*S. aureus* 5026	16	256	1
*S. aureus* 5027	16	64	1
*S. aureus* 5028	16	64	1
*S. aureus* 5029	16	64	1
*S. aureus* 3211	8	128	1
*S. aureus* 3304	16	128	1
*S. aureus* 2798	8	128	1
*Bacillus subtilis* 168	32	128	0.25
*Escherichia coli* ATCC 35218	>128	128	—
*Pseudomonas aeruginosa* ATCC 27853	>128	256	—

*MIC* minimum inhibitory concentration

^a^—, not determined

To evaluate the bactericidal behavior of EF60, the MBC was determined for the three standard strains and the two clinical isolates of *S. aureus*. The control agent, vancomycin, yielded MBC/MIC ratios of 1–2 against all of the strains tested (Table 3), indicative of bactericidal antistaphylococcal behavior. Interestingly, EF60 exhibited MBC/MIC ratios of 1–2 against ATCC 21923 and clinical isolate 3304, but it yielded MBC/MIC ratios >8 against ATCC 25923, ATCC 43300, and clinical isolate 3211 (Table 3). In accordance with the standards of the Clinical and Laboratory Standards Institute [30], an MBC/MIC ratio of 1–2 is indicative of bactericidal behavior, while a corresponding MBC/MIC ratio ≥8 is indicative of bacteriostatic behavior. Thus, EF60 is bactericidal against *S. aureus* 3304 and ATCC 29213 and bacteriostatic against *S. aureus* 3211, ATCC 25923, and ATCC 43300. Similarly, berberine was bacteriostatic against *S. aureus* ATCC 25923 and ATCC 29213 and bactericidal against *S. aureus* 3211, 3304, and ATCC 43300.

**Table 3:** The MBC of EF60 against standard strains and clinical isolates

Strain and compound	MIC (μg/mL)	MBC (μg/mL)	MBC/MIC
*S. aureus* ATCC 25923			
EF60	32	>256	>8
Berberine	128	>512	>4
Vancomycin	2	2	1
*S. aureus* ATCC 29213			
EF60	16	32	2
Berberine	64	>512	>8
Vancomycin	1	2	2
*S. aureus* ATCC 43300			
EF60	8	>128	>8
Berberine	128	128	1
Vancomycin	2	2	1
*S. aureus* 3211			
EF60	8	>128	>16
Berberine	128	256	2
Vancomycin	1	2	2
*S. aureus* 3304			
EF60	16	32	2
Berberine	128	128	1
Vancomycin	1	1	1

*MIC* minimum inhibitory concentration

*MBC* minimum bactericidal concentration

**Fig. 1:**
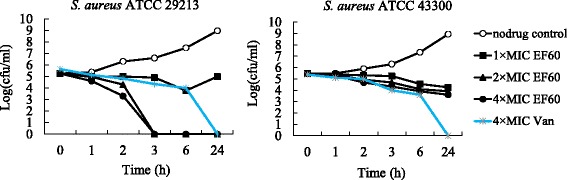
Time-kill curves for *Staphylococcus aureus* ATCC 29213 and 43300. Each data point reflects the average of two independent measurements. The curves are viable cell concentrations plotted against time. Open circles, nondrug control; closed squares, 1× MIC of EF60; closed triangles, 2× MIC of EF60; closed circles, 4× MIC of EF60; asterisk, 4× MIC of vancomycin. MIC, minimum inhibitory concentration

To further examine the bactericidal/bacteriostatic activities of EF60 against *S. aureus* with regard to MBC/MIC ratios, killing experiments against ATCC 29213 and 43300 were performed. As shown in Fig. 1, EF60 produced >3 logs of kill against ATCC 29213 at concentrations of ≥2 × the MIC. However, less than 2 logarithmic units of killing were observed, even at 4× the MIC of EF60, against ATCC 43300. This observation is consistent with the bactericidal/bacteriostatic behavior revealed by the MBC/MIC analysis described above. It is worth mentioning that at concentrations of ≥2× the MIC, the killing kinetics of EF60 against ATCC 29213 are faster than those of vancomycin.

### Effect of pH and inoculum size on MICs of EF60

Table 4 summarized the MICs for the two strains of *S. aureus* obtained at different pH and inoculum concentrations. There was only a twofold increase in the MICs of EF60 for ATCC 29213 and 43300, from 16 and 8 μg/mL at pH 5 to 32 and 16 μg/mL at pH 9, respectively. Similar reduction potency was observed when the inoculum sizes changed from 10^3^ to 10^7^ CFU/mL. Generally, both the pH and inoculum sizes did not significantly affect the MICs of EF60 against the two strains of *S. aureus* under the tested conditions.

**Table 4:** The effect of changes in the pH medium and the inoculum size on EF60 activity

Influence factor		MIC (μg/mL)	
		*S. aureus* ATCC 29213	*S. aureus* ATCC 43300
pH of medium	5	16	8
	7	16	8
	9	32	16
Inoculum size (CFU/mL)	10^3^	16	8
	10^5^	16	8
	10^7^	32	16

*MIC* minimum inhibitory concentration

### Postantibiotic effect

The PAE of EF60 on the reference strains was determined and is shown in Table 5. A PAE of > 1.5 h was observed on strain ATCC 29213 for all tested concentrations of EF60, compared with the maximum PAE of 0.38 h for strain 43300 after exposure to 64 μg/mL of EF60. Although the PAE of EF60 increased with increasing concentrations of the drug, the dose-dependent PAE on *S. aureus* from 1 to 4× the MIC of EF60 was not significant (*P* > 0.05).

**Table 5:** PAE of EF60 against *Staphylococcus aureus* strains^a^

Drug concentration in μg/mL	PAE (h) mean ± SD	
	*S. aureus* ATCC 29213	*S. aureus* ATCC 43300
16 (1× MIC)	1.53 ± 0.08	0.28 ± 0.04
32 (2× MIC)	1.87 ± 0.02	0.30 ± 0.06
64 (4× MIC)	2.00 ± 0.05	0.38 ± 0.06

^a^Approximately 10^5^ CFU/mL was exposed to different concentrations of EF60 for 1 h

### Cytotoxicity

The cytotoxicity of EF60 against mammalian cells was tested by hemolysis and MTT assays. No hemolytic activity was observed against human erythrocytes when the concentration of EF60 reached 160 μg/mL. At this concentration, EF60 effectively inhibited the growth of all of the Gram-positive bacteria tested, including MRSA. However, EF60 was minimally toxic to HUVEC, with an IC_50_ of 75 μg/mL, indicating a different cytotoxicity against cell lines derived from diverse tissues. Future studies with additional human cell lines will be done to evaluate the toxicity of EF60.

## Discussion

In this study, a plant fraction (EF60) from the ethanol extract of fibrous roots of *B. striata* pseudobulbs was isolated and characterized. EF60 was eluted from a polyamide resin column with 60% ethanol and found to be rich in phenanthrenes. Antimicrobial activity tests demonstrated that EF60 had good activity against Gram-positive bacteria, including *S. aureus* clinical isolates and MRSA. Interestingly, the MICs of EF60 against all tested *S. aureus* strains were 8–64 μg/mL and lower than those of berberine (Table 2), which is a famous natural antibiotic in China. On the basis of the MIC values, which are below 100 μg/mL for the fraction against Gram-positive bacteria, EF60 is regarded as a significantly active antibacterial agent and deserve our full attention [31, 32].

Many antibiotics used in clinical, such as penicillin, vancomycin, and daptomycin, exhibit fast and bactericidal effects. Some, such as erythromycin and tigecycline, however, are bacteriostatic rather than bactericidal. To evaluate the bactericidal behavior of EF60, the MBC was determined for five *S. aureus* strains*.* As shown in Table 3, against 3 of these strains EF60 showed only bacteriostatic activities. With the other 2 strains, bactericidal activities of EF60 were observed. Similar phenomena were also observed for berberine and in other studies [33]. The bactericidal/bacteriostatic behavior of EF60 was confirmed by time-kill assays (Fig. 1).

It was reported that the pH of culture medium or inoculum size had an effect on the antibacterial activities of some antibiotics [25, 31]. For example, amifloxacin was more active against *Staphylococcus saprophyticus* at pH 6.0 than at 7.0 [25], while the oil of *Cedrus deudora* had most active at pH 9 [31]. In the present study, variations in the pH of the medium or inoculum density had no significant effect on the activity of EF60 (Table 4), indicated this plant fraction had a high stability when susceptibility test conditions were modified.

PAE is the phenomenon of suppression of bacterial growth after a short exposure to antimicrobial agents [26, 34]. It is an important parameter of antibiotic action, and provides reference data for designing antibiotic dosage regimens. Previous studies indicated that many test antibiotics had a persistent inhibition of bacterial growth after a brief antimicrobial exposure to microorganisms [35–37], while some drugs had insignificant PAE [34]. Our study showed that the PAE was related with both the concentration of EF60 and test strains: for example, the PAEs for Strain ATCC 29213 were 1.53 h after exposure to 16 μg/mL and 2.0 h after exposure to 64 μg/mL, but the PAE for Strain ATCC 43300 was 0.38 h after exposure to 64 μg/mL (Table 5).

Herbal drugs are often claimed to be nontoxic or low toxic, but this is not always the case, especially for certain plant extracts and phytochemicals [32]. It is important to measure the toxicity of new antimicrobial agents to cell lines and animals. The in vitro cytotoxicity assay indicated that EF60 was minimally toxic to HUVEC with an IC_50_ of 75 μg/mL. More studies with additional human cell lines and animals should be done to evaluate the toxicity of EF60.

## Conclusion

In conclusion, EF60, a plant fraction rich in phenanthrenes, has a potent activity against Gram-positive bacteria, including MRSA and *S. aureus* clinical isolates, which represents the most frequent cause of complicated skin and soft tissue infections worldwide [38]. This antimicrobial activity of EF60 seems to have a direct correlation to the traditional use of the herb for curing skin cracks and abscesses. Our study revealed that EF60 may be applied to the development of natural antibacterial products. However, more studies on the in vivo antimicrobial activity, bioavailability, and mechanism of action of EF60 are needed to be conducted.

## Additional file

Additional file 1: Figure S1. HPLC analysis of standard sample and EF60. **Table S1.** The retention times of standard samples and compounds of EF60. **Figure S2.** UV spectrum of standard samples and compounds 1–8 of EF60. (DOCX 427 kb)

## Abbreviations

DMSO: Dimethyl sulfoxide
HPLC: High-pressure liquid chromatography
HUVEC: Human umbilical vein endothelial cells
MBC: Minimum bactericidal concentration
MIC: Minimum inhibitory concentration
MRSA: Methicillin-resistant Staphylococcus aureus
MTT: 3-(4,5-dimethylthiazol-2-yl)-2,5-diphenyltetrazolium bromide
PAE: Postantibiotic effect
VAN: Vancomycin
