# Supplementary material for: The anti-Staphylococcus aureus activity of the phenanthrene fraction from fibrous roots of Bletilla striata
Source: BMC Complement Altern Med. 2016 Nov 29;16:491. doi: 10.1186/s12906-016-1488-z (PMC5129615; doi:10.1186/s12906-016-1488-z)
Supplement: Additional file 1: Figure S1. — HPLC analysis of standard sample and EF60. Table S1. The retention times of standard samples and compounds of EF60. Figure S2. UV spectrum of standard samples and compounds 1–8 of EF60. (DOCX 427 kb) [file 12906_2016_1488_MOESM1_ESM.docx]

**The Anti-*Staphylococcus aureus* Activity of the Phenanthrene Fraction from Fibrous Roots of *Bletilla striata***

Jing-Jing Guo^1,2^, Bin-Ling Dai^1^, Ni-Pi Chen^1^, Li-Xia Jin^1^, Fu-Sheng Jiang^1^, Zhi-Shan Ding^1^ and Chao-Dong Qian^1^*

^1^ Zhejiang Chinese Medical University, Hangzhou, Zhejiang province, China

^2^ Shaoxing Central Hospital, Shaoxing, Zhejiang province, China

*Corresponding authors

E-mail: [qian201@163.com](mailto:qian201@163.com). Tel.: +86-571-86613666.

**[See Main Manuscript]**

The compositions of EF60 were separated by HPLC-DAD, and more than 8 peaks were detected (**Fig. S1**). Among them, 4,5,7,8, were found to be the major peaks, and exhibited the same retention time with biphenanthrene standard sample S2, S3, S4, and S7, respectively (**Table S1**). In addition, standard samples S1-S7 as well as compounds 1-8 of EF60 exhibited typical UV spectrum of biphenanthrene with absorption at about 260, 208, and 305 nm (**Fig. S2**). Thus, it is reasonable to speculate that EF60 was found to be rich in phenanthrenes. Standard samples were isolated from the Fibrous Roots of *Bletilla striata* and were well characterized [1,2]. S1: 4,4’,7,7’-tetrahydro-2,2’-dimethoxy-1,1’-biphenanthrene; S2: 4,4’,7,7’-tetrahydro- 2,2’, 8-trimethoxy -1,1’-biphenanthrene; S3: 4,4’,7,7’-tetrahydro-2,2’,8,8’- tetramethoxy-11’- biphenanthrene; S4: 4,4’,7-tetrahydro-2,2’,7’-trimethoxy-1,1’- biphenanthrene; S5: 4,8,4′,8′-tetramethoxy(1,1′-biphenanthrene)-2,7,2′,7′-tetrol; S6: 4,7,3′,5′-tetramethoxy-9′,10′-dihydro(1,1′-biphenanthrene)-2,2′,7′-triol; S7: 4,7,7′- trimethoxy-9′,10′-dihydro (1,3′-biphenanthrene)-2,2′,5′-triol.


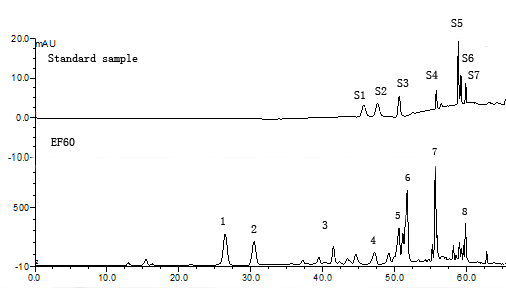


**Fig. S1** HPLC analysis of standard sample and EF60. Samples were analyzed by HPLC on a Dionex C18 reversed-phase column (250 × 4.6 mm, 5μm). The mobile phase was composed of an acetonitrile-water (0.1% formic acid) (30:70) mixture and programmed using a isocratic elution for 25min, a linear gradient from 30% to 40% for 10 min, another isocratic elution for 10 min, and a last linear gradient from 40% to 100% for 25 min. The UV detector was set to 210, 260 and 280 nm, and the flow rate was 1 mL/min.

**Table S1** The retention times of standard samples and compounds of EF60

| standards | Ret.Time (min) | Compounds of EF 60 | Ret.Time (min) |
| --- | --- | --- | --- |
| S2 | 47.590 | 4 | 47.185 |
| S3 | 50.597 | 5 | 50.547 |
| S4 | 55.752 | 7 | 55.615 |
| S7 | 59.840 | 8 | 59.803 |


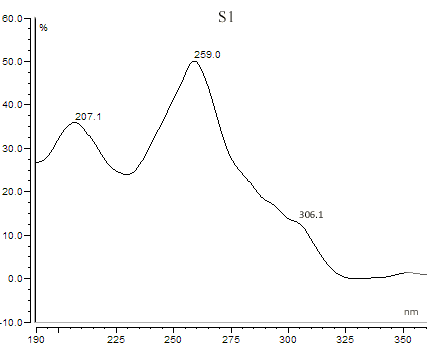


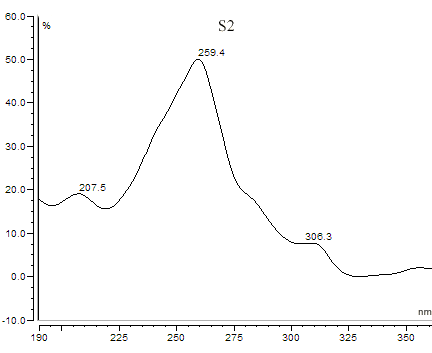


**Fig. S2 [continue].**


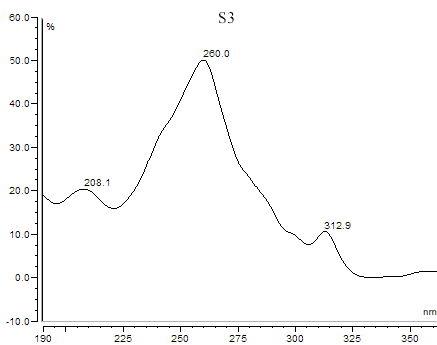


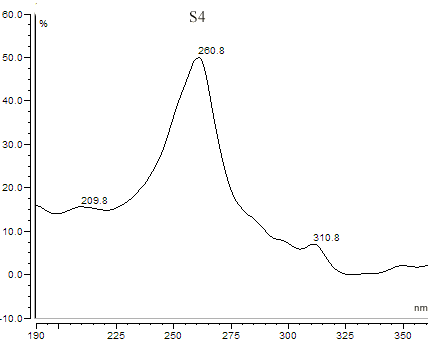


**Fig. S2 [continue].**


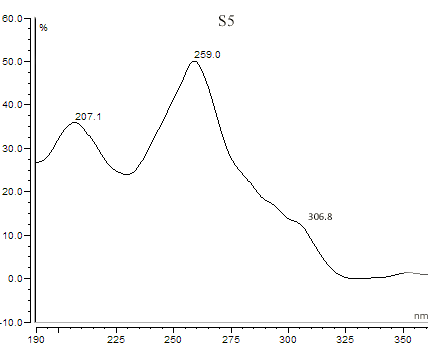


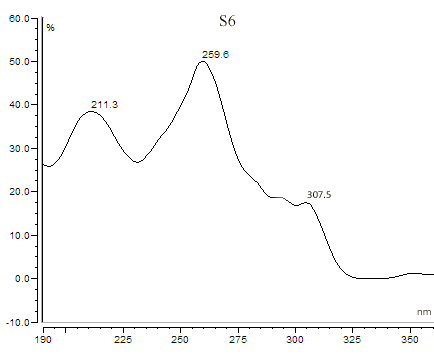


**Fig. S2 [continue].**


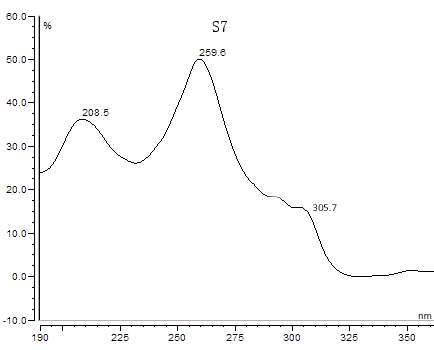


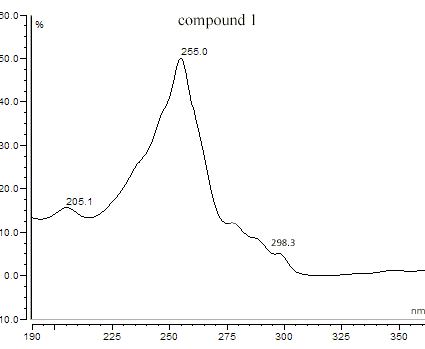


**Fig. S2 [continue].**


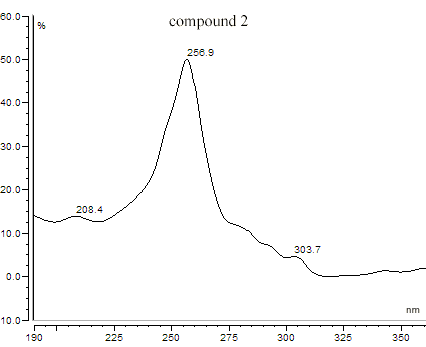


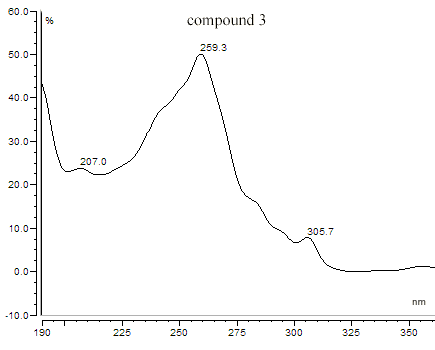


**Fig. S2 [continue].**


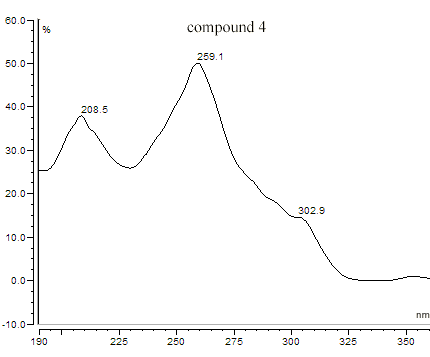


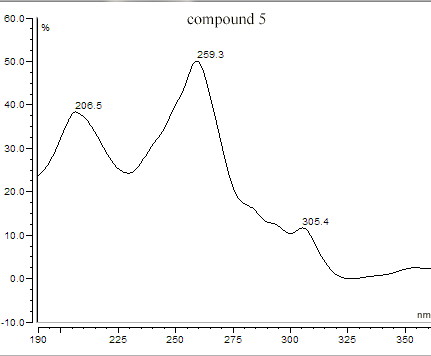


**Fig. S2 [continue].**


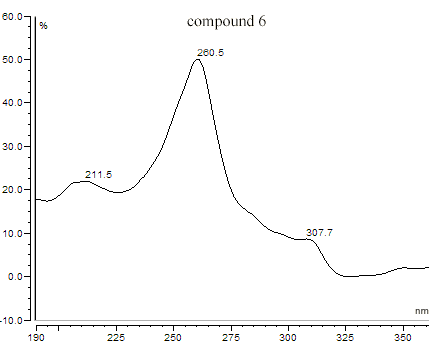


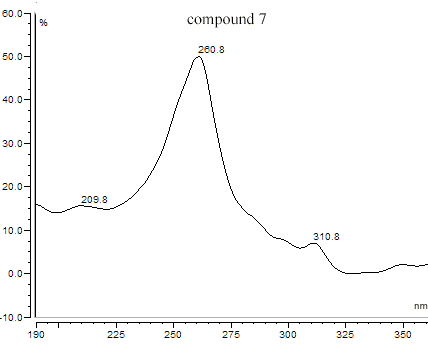


**Fig. S2 [continue].**

**
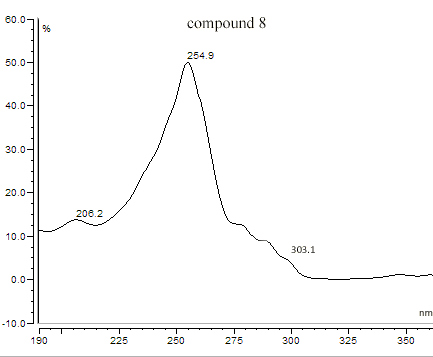
**

**Fig. S2 [end].**

**Fig. S2** UV spectrum of standard samples and compounds 1-8 of EF60

**References**

[1] Qian C-D, Jiang F-S, Yu H-S, Shen Y, Fu Y-H,et al. Antibacterial Biphenanthrenes from the Fibrous Roots of *Bletilla striata*. J Nat Prod. 2015; 78:939−943.

[2] Yong Shen. Studies on chemical constituents and antitumor activities of the fibrous root part of *Bletilla striata* (Thunb.) Reichb.f [D]*.* Hangzhou: Zhejiang Chinese Medical University, 2014.
